# Utility and Implementation of the Distress Thermometer for Cancer Patients: A Cross-Sectional Study From Saudi Arabia

**DOI:** 10.7759/cureus.57187

**Published:** 2024-03-29

**Authors:** Mohammed Alghamdi, Fadi Masharqa, Abdulrahman Alsultan, Sarah Sewaralthahab, Omar Abdelwahab, Sherif Mohamed, Nashwa Abd El-Aziz, Fahad D Alosaimi

**Affiliations:** 1 Department of Medical Oncology, King Saud University Medical City, King Saud University, Riyadh, SAU; 2 Department of Hematology/Oncology, King Saud University Medical City, King Saud University, Riyadh, SAU; 3 Department of Pediatric Oncology, King Saud University Medical City, King Saud University, Riyadh, SAU; 4 Department of Hematology and Medical Oncology, King Saud University Medical City, King Saud University, Riyadh, SAU; 5 College of Medicine, Alfaisal University, Riyadh, SAU; 6 Department of Chest Diseases and Tuberculosis, Faculty of Medicine, Assiut University, Assiut, EGY; 7 Department of Internal Medicine, Sultan Bin Abdulaziz Humanitarian City, Riyadh, SAU; 8 Department of Medical Oncology, South Egypt Cancer Institute, Assiut University, Assiut, EGY; 9 Department of Medical Oncology, National Blood and Cancer Center, Riyadh, SAU; 10 Department of Psychiatry, King Saud University, Riyadh, SAU

**Keywords:** distress thermometer, saudi arabia, cross-sectional, implementation, emotional distress, cancer patients

## Abstract

Background

Cancer patients suffer from variable degrees of distress. The distress thermometer (DT) is a valuable tool for screening those patients for distress. Few studies have addressed the utility of DT in screening cancer patients in Saudi Arabia. We aimed to measure the distress level of adult cancer patients utilizing the DT and identify the appropriate measures and interventions required to improve this population's well-being.

Methods

This cross-sectional study was carried out at the oncology center of King Saud University Medical City (KSUMC), Riyadh, Saudi Arabia. Enrollment criteria were Saudi adults (≥14 years old), with a diagnosis of cancer, who gave informed consent. They were screened for distress using the DT and its associated problem list (PL). A workflow for a psycho-oncology supportive program was suggested.

Results

Using DT at a cut-off score of ≥4, 22% of patients had significant distress. The most frequent problems reported were loss/change of physical activity, swelling/edema, change in eating, family health problems, and child care. The multivariable binary regression analysis showed that sadness, depression, worry/anxiety, fear, loss of interest, change in appearance, taking care of myself, swelling/edema, and memory/concentration problems were independent factors for significant distress in our cohort. The suggested workflow could effectively be implemented among cancer patients.

Conclusion

The current study's findings support previous reports concerning the utility of DT in screening cancer patients for distress. A considerable number of Saudi cancer patients suffered from significant distress, which was significantly related to the emotional, spiritual, social, and religious aspects of their problems. We suggested a workflow by which cancer centers can implement DT screening after developing a plan for timely distress evaluation, with further proper management and referrals accordingly. Additional studies are warranted.

## Introduction

Newly diagnosed cancer cases in Saudi Arabia were 20131 in 2018 [1]. The most common malignancy among Saudi females was breast cancer, whereas colorectal cancer was ranked first and third for Saudi men and women, respectively [1].

The term "distress" was adopted by the well-known cancer network, i.e., the National Comprehensive Cancer Network (NCCN), as an emotionally unpleasant psychological, spiritual, and social experience that might affect the cancer patient’s capability to cope with his/her disease, its symptoms, as well as its therapies [2].

It has been indicated that receiving a cancer diagnosis and undergoing multimodal therapy increases the risk of experiencing emotional distress (ED) and mental comorbidities. Thus, the quality of life of cancer patients can be significantly affected in various ways when they experience unpleasant therapy side effects and abnormal levels of physical symptom burden [3-5]. Thus, screening cancer patients for any distress is thought to be an effective way to provide them with timely initiated support, especially those who have priority for that [6-9]. Therefore, it is unsurprising that several international regulatory organizations have strongly recommended “routine” screening and proper distress management.

The Multinational Association of Supportive Care in Cancer (MASCC) defines supportive care as "the prevention and management of the adverse effects of cancer and its treatment" [10]. Change in name (from palliative care to supportive care/oncology) results in more and earlier referrals to hospital-based services. Also, it will be more accepted by patients and families as an integral part of cancer care from day one of the cancer journey [11]. An important part of the supportive oncology program is applying the "stepped care approach" [12].

The distress thermometer (DT) is the ideal screening tool because it is a quick and valid tool for recognizing, diagnosing, and promptly managing distress in cancer patients. The original NCCN has been successfully translated into several languages, including Arabic [13].

Few studies have evaluated the usefulness of DT among Saudi cancer patients [4,14]. Therefore, the current study aims to measure the distress level of adult cancer patients at a tertiary referral oncology center utilizing the NCCN-adopted DT and identify the appropriate measures and interventions required to improve this population's well-being.

## Materials and methods

Study design and population

The current cross-sectional study was carried out at King Saud University Medical City (KSUMC), King Saud University, Kingdom of Saudi Arabia. The Institutional Review Board approved the study. Newly diagnosed cancer patients were prospectively enrolled in the study. Enrollment criteria were Saudi adults (≥14 years old) who had a confirmed diagnosis of cancer and who gave informed consent. Exclusion criteria were patients with a history of or who were already undergoing management for psychiatric illness.

Social and demographic data

The patient’s electronic medical records were used to retrieve the patient’s sociodemographic and clinical features. Sociodemographic data were collected, including marital status, gender, age, level of education, oncologic performance status, type and stage of cancer, and the therapeutic modalities used.

Distress thermometer

The current study used the recently published and validated Arabic version of the NCCN-adopted DT [13] with a cut-off score of ≥4. DT screening was carried out for patients with oncologic malignancy cancer at their first outpatient visit or inpatient admission.

Consequently, to check whether they had any problems listed during the questionnaire, the patients were requested to fill in the DT-associated problem list (PL) [2,13]. For illiterate patients, a research assistant helped them to rate their distress and fill in the PL. This PL consisted of 36 problems of five categories: family, emotional, spiritual/religious, practical, and physical issues. To identify the nature of the distress and related factors, correlations between the PL and DT were conducted.

Implementing a psycho-oncology program

A multidisciplinary expert committee reviewed the relevant literature, developed a policy, and suggested the following objectives of the Supportive Oncology Program at KSUMC: (1) to provide full access to supportive care for all oncology patients and their families followed up at KSUMC; (2) to initiate and maintain a patient-centered, compassionate care culture; (3) to work in partnership with all KSUMC clinics, divisions, and departments; (4) to streamline the referral process (when indicated) to give patients timely access to proper supportive care; (5) advanced understanding and proper knowledge relating to psychosocial and palliative interventions and modalities of care; (6) to provide essential education opportunities for healthcare professionals and related disciplines.

**Figure 1:**
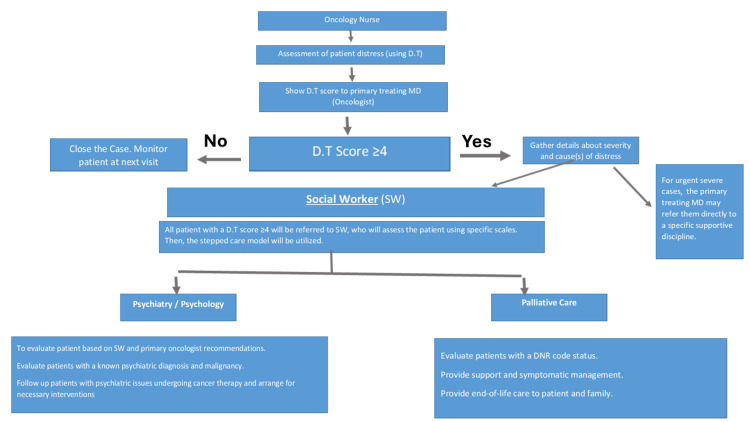
Workflow for psycho-oncology support of cancer patients DT: distress thermometer; MD: Doctor of Medicine; DNR: do not resuscitate.

All cancer care providers, including oncologists, palliative care physicians, psychiatrists, social workers, psychologists, nurses, dietitians, occupational therapists, speech-language pathologists, spiritual care practitioners, physiotherapists, healthcare administrators, and community societies, participated in a multidisciplinary approach to be sure that patients with cancer receive the standard of supportive oncology care. A workflow was suggested for eligible cancer patients (Figure 1).

Statistical analysis

Data analysis was carried out via the use of SPSS version 25 (IBM Corp., Armonk, NY). The binary logistic regression test was carried out to investigate any possible association between the sociodemographic and clinical variables on one hand and the DT at cut-off scores of 4 on the other hand. The multivariable regression analysis was utilized to examine the association between the cut-off scores and individual items in the PL. All p-values were two-tailed. The p-value < 0.05 was considered statistically significant.

## Results

Clinical and sociodemographic characteristics

A total of 100 patients were enrolled in the current study, with a mean age of 53.23 ± 13.3 years. Males and females constituted 34% and 66% of the participants, respectively.

Different types of malignancies were included. The most common were gastrointestinal tumors (41%), followed by breast cancer (32%), genitourinary (14%), and bone tumors (5%). Of the patients, 2% had stage I, whereas 8%, 29%, and 61% had stage II, III, and IV, respectively (Table 1).

**Table 1:** Univariate correlates of DT among the studied cohort DT: distress thermometer; DTC: distress thermometer cutoff.

Item	Total (n = 100)	DTC ≥ 4 (n = 22)	DTC < 4 (n = 78)	P-value
Age (years)	53.23 ± 13.3	56.86 ± 10.5	52.21 ± 13.9	0.097
Sex (male/female)	34/66	6/16	28/50	0.451
Tumor site				
Breast	32 (32%)	7 (31.8%)	25 (32.1%)	0.018
Gastrointestinal	41 (41%)	10 (45.5%)	31 (39.7%)	
Genitourinary	14 (14%)	1 (4.5%)	13 (16.7%)	
Bone	5 (5%)	4 (18.2%)	1 (1.3%)	
Others	8 (8%)	0 (0%)	8 (10.2%)	
Stage at Interview				
Stage I	2 (2%)	0 (0%)	2 (2.6%)	0.302
Stage II	8 (8%)	3 (13.6%)	5 (6.4%)	
Stage III	29 (29%)	3 (13.6%)	26 (33.3%)	
Stage IV	61 (61%)	16 (72.8%)	45 (57.7%)	
Treatment received				
Chemotherapy	69 (69%)	18 (81.8%)	51 (65.4%)	0.105
Hormonal	13 (13%)	2 (9.1%)	11 (14.1%)	
Biological	4 (4%)	0 (0%)	4 (5.1%)	
Chemo-bio	12 (12%)	2 (9.1%)	10 (12.8%)	
Hormonal-bio	2 (2%)	0 (0%)	2 (2.6%)	

Data from the distress thermometer and its associated problem list

Of the patients, 22% experienced statistically significant distress (DT cut-off score ≥ 4), with an encountered patient’ average DT score of 3.7. There were no significant differences between patients with significant distress and those without substantial distress about age, gender, stage of the disease, and therapeutic modality (Table 1).

The most frequently encountered problems in the domain of the PL were in this descending order: loss/change of physical activity, swelling/edema, change in eating, family health problems, and child care.

Relationship between significant DT score and different PL items

It was observed that there was a statistically significant correlation between the DT score of ≥4 and most of the PL items, including child care, fear, anger, sadness, worry/anxiety, loss of interest, spiritual/religious concern, change in appearance, taking care of myself, swelling/edema, loss/change of physical activity, memory/concentration, nasal dryness, and sexual health (Table 2).

**Table 2:** Univariate correlates of DT-associated PL among the studied cohort DT: distress thermometer; DTC: distress thermometer cutoff; PL: problem list.

Problem list	Total (n = 100)	DTC ≥ 4 (n = 22)	DTC < 4 (n = 78)	P-value
Child care	76 (76%)	22 (100%)	54 (69.2%)	0.003
Housing	12 (12%)	3 (13.6%)	9 (11.5%)	0.723
Insurance/finance	5 (5%)	0 (0%)	5 (6.4%)	0.583
Work/school	13 (13%)	3 (13.6%)	10 (12.8%)	0.487
Treatment decision	12 (12%)	4 (18.2%)	8 (10.3%)	0.456
Relation (children)	14 (14%)	4 (18.2%)	10 (12.8%)	0.369
Relation (partner/spouse)	2 (2%)	1 (4.5%)	1 (1.3%)	0.393
Ability to have children	1 (1%)	1 (4.5%)	0 (0%)	0.220
Family health problem (depression)	71 (71%)	18 (81.8%)	53 (67.9%)	0.205
Fear	13 (13%)	8 (36.4%)	5 (6.4%)	0.001
Anger	13 (13%)	7 (31.8%)	6 (7.7%)	0.007
Sadness	12 (12%)	8 (36.4%)	4 (5.1%)	<0.001
Worry/anxiety	18 (18%)	13 (59.1%)	5 (6.4%)	<0.001
Loss of interest	6 (6%)	5 (22.7%)	1 (1.3%)	0.002
Spiritual/religious concern	2 (2%)	2 (9.1%)	0 (0%)	0.047
Change in appearance	32 (32%)	13 (59.1%)	19 (24.4%)	0.009
Taking care of myself	20 (20%)	9 (40.9%)	11 (14.1%)	0.013
Change in urination	1 (1%)	1 (4.5%)	0 (0%)	0.220
Diarrhea	24 (24%)	8 (36.4%)	16 (20.5%)	0.124
Change in eating	50 (50%)	14 (63.6%)	36 (46.2%)	0.148
Swelling/edema	51 (32%)	17 (77.3%)	34 (43.6%)	0.005
Loss/change of physical activity	54 (32%)	17 (77.3%)	37 (47.4%)	0.013
Memory/concentration problem	6 (6%)	5 (22.7%)	1 (1.3%)	0.002
Mouth ulcer	8 (8%)	2 (9.1%)	6 (7.7%)	0.562
Nasal dryness	24 (24%)	11 (50%)	13 (16.7%)	0.003
Sexual health	9 (9%)	3 (13.6%)	6 (7.7%)	0.005
Skin dryness	16 (16%)	5 (22.7%)	11 (14.1%)	0.252

The multivariable binary regression analysis confirmed that fear, depression, worry/anxiety, loss of interest, sadness, change in appearance, taking care of myself, swelling/edema, and memory/concentration problems were independent factors for significant distress in our cohort (Table 3).

**Table 3:** Independent predictors of DT: binary regression analysis DT: distress thermometer.

Item	OR (95% CI)	P-value
Age (years)	1.014 (0.948 – 1.085)	0.682
Sex (male)	1.682 (0.577 – 4.902)	0.341
Depression	2.297 (1.021 – 6.788)	0.044
Fear	8.343 (2.387 – 19.271)	0.001
Sadness	7.368 (1.515 – 15.831)	0.013
Worry/anxiety	17.434 (4.713 – 34.489)	<0.001
Loss of interest	13.191 (1.276 – 33.372)	0.030
Change in appearance	2.421 (1.081 – 8.646)	0.014
Taking care of myself	4.417 (1.457 – 12.202)	0.008
Swelling/edema	4.401 (1.475 – 13.126)	0.011
Memory/concentration problem	22.647 (2.484 – 46.515)	0.006

The psycho-oncology program

With the implementation of the workflow as mentioned above, cancer patients with a DT score ≥4 were referred to the gatekeeper of the supportive oncology program, a social worker (SW). SW will assess the patient thoroughly using specific scales. They will also evaluate the family. Then, accordingly, we will use the stepped care model to help the patient. For instance, SW will determine the appropriate program (individual vs. group), provide social or psychological support, and prepare social reports to submit to supporting associations. As part of the supportive oncology program, the SW will make sure that all patients receive care with the minimal necessary time with the trained clinician but are monitored systematically to "step up" to more intensive treatment if they do not achieve sufficient health gain with low-intensity treatment. Among the healthcare providers, the SW may consult different specialties (e.g., oncologists, palliative care physicians, psychiatrists, psychologists, occupational therapists, physiotherapists, and spiritual care practitioners) and re-providers in the community (Figure 1).

## Discussion

The current study's findings support previously published reports on Saudi Arabian cancer patients [4,13,14] and confirm the utility of DT for screening cancer patients for ED [2,3,5-9]. Using the DT, we identified that 22% of our cancer patients had significant distress, which was significantly related only to the tumor site. The lower number of enrolled subjects may explain this lower distress prevalence.

It was beneficial that the NCCN adopted the DT as an instrument to identify sources of distress after cancer diagnosis [2]. Moreover, the DT-associated PL clearly provides a comprehensive list of categories and covers almost all aspects that might contribute to distress among cancer patients [2-8]. The DT appears so simple, yet it covers most, if not all, problems that might be challenging to any study population and on a worldwide scale.

The site of cancer can be an important predictor of overall distress, and a significant correlation was found between these two variables. Evidence shows that emotional suffering may have some predictive capacity for some cancer presentations [15].

The frequently reported problems in the practical domain of the PL were loss or change of physical activity, swelling or edema, change in eating, family health problems, and child care. These findings agree with those reported by Alosaimi and colleagues [13], which could be explained by the cultural demographic similarities among Saudi populations. In daily practice, these encountered problems represent the most challenging ones for oncologists/psycho-oncologists.

Cancer diagnosis may result in various emotions, including depression, lack of interest, anxiety, or anger. Even after cancer has gone into remission, the patient may experience frustrating side symptoms or worry that it will return. Following a cancer diagnosis, it is expected to have bouts of melancholy. On the other hand, prolonged feelings of melancholy can develop into depression, which is a more dangerous stage. These factors should be taken into consideration for psychological counseling of cancer patients, which is an essential aspect of successful cancer management [3,4,13,14].

The results of this study highlight the importance of early screening cancer patients (i.e., at their initial diagnosis with cancer) for distress. We have observed that participants who scored ≥4 on the DT had significantly extra problems in different aspects compared to patients who scored below this cut-off score.

Depression, fear, sadness, worry/anxiety, loss of interest, change in appearance, taking care of myself, swelling/edema, and memory/concentration problems were independent factors for significant distress in our cohort.

People coping with life-threatening illnesses, such as cancer, may be at a greater risk of experiencing spiritual and/or religious distress. Patients may find it easier to deal with the difficulties associated with their sickness if their spiritual needs are met. Because spirituality is such an essential part of person-centered and holistic care, the treatment plan for each cancer patient should include some spiritual intervention, whether it be prayer, meditation, or something else [13,14,16]. The religious beliefs of the Saudi population are firm. Thus, we found the association of spiritual and religious concerns positively correlated with distress levels. This phenomenon is termed spiritual distress/religious distress [16].

Overall, in the current study, we have identified that the critical stress determinants are the prime determinants of neurological health and cognitive functioning in cancer patients. The early evaluation of the essential stress levels in those patients can help identify the measures that can improve psychological well-being and mental health [8-10].

The current study's workflow has the potential to serve as a proactive model to improve the management of cancer patients in a step-wise fashion, taking into consideration personalized care (Figure 1).

The DT will assess the patient's overall discomfort level as the first step in the evaluation process. The overall distress score will be determined, and accordingly, further management will be implemented. The psychologist/psychiatrist will be concerned with evaluating the patient based on SW and oncologist recommendations, evaluating patients with a known combined psychiatric diagnosis and malignancy, and following up on patients with psychiatric issues undergoing cancer therapy. On the other hand, the palliative care specialist will be concerned with evaluating patients with a do-not-resuscitate (DNR) code status, providing support and symptomatic management, and providing end-of-life care to dedicated patients and their families. This proactive model will help provide the best supportive measures for cancer patients and their caregivers.

Lastly, keeping in view the high religious and spiritual morals and strong beliefs of the Saudi population, the spiritual and religious reprogramming of the patient's beliefs can be very helpful in reducing overall distress. Being a cross-sectional study does not guarantee that it has no limitations. Possible limitations include a low number of enrolled subjects and a single-center experience. Further, more extensive studies are warranted.

## Conclusions

The current study's findings support previous reports concerning the utility of DT in screening cancer patients for distress. A considerable number of Saudi cancer patients suffered from significant distress, which was significantly related to the emotional, spiritual, social, and religious aspects of their problems. We suggested a workflow through which cancer centers can implement DT screening after developing a plan for timely distress evaluation, with further proper management and referrals accordingly. Further research is warranted to confirm this workflow implementation.
